# Systematic review of the association between dietary patterns and perinatal anxiety and depression

**DOI:** 10.1186/s12884-019-2367-7

**Published:** 2019-06-24

**Authors:** David Franciole Oliveira Silva, Ricardo Ney Cobucci, Ana Katherine Gonçalves, Severina Carla Vieira Cunha Lima

**Affiliations:** 1 Postgraduate Program in Nutrition, Federal University of Rio Grande do Norte – UFRN, Natal, RN Brazil; 2 Postgraduate Program in Biotechnology, Potiguar University – UnP, Natal, RN Brazil; 3 Department of Obstetrics and Gynecology, Federal University of Rio Grande do Norte - UFRN, Natal, RN Brazil; 4 Department of Nutrition, Federal University of Rio Grande do Norte – UFRN, Avenida Senador Salgado Filho, 3.000, Campus Universitario, Lagoa Nova, Natal, RN Brazil

**Keywords:** Dietary patterns, Anxiety, Depression, Pregnancy, Postpartum, PAAD

## Abstract

**Background:**

Specific dietary factors contribute to greater risks of prenatal and postpartum anxiety and depression. This study aimed to systematically review and assess the evidence regarding the association between dietary patterns and perinatal anxiety and depression (PAAD).

**Methods:**

A systematic search of the Latin American & Caribbean Health Sciences Literature (LILACS), PubMed, and Scopus databases for cross-sectional and cohort studies through April 2019 was conducted. The methodological quality of the studies was assessed using the Newcastle-Ottawa scale (NOS) and the Grading of Recommendations Assessment, Development and Evaluation (GRADE) framework was used to assess the quality of evidence.

**Results:**

Ten studies (six cohort and four cross-sectional) were included. All studies had good methodological quality. In these studies, the Western (*n* = 10), healthy (*n* = 9), and traditional (*n* = 7) dietary patterns were examined. The healthy pattern was inversely associated with prenatal and postpartum anxiety and prenatal depression. The traditional Japanese dietary pattern, the traditional Indian-confinement dietary pattern, the United Kingdom traditional dietary and the traditional Brazilian dietary pattern were associated with a lower risk of prenatal depression, postpartum depression, prenatal anxiety and postpartum anxiety, respectively. There was no significant association between a Western dietary pattern and PAAD. The GRADE assessment suggested that the quality of the evidence was very low to low across all outcomes owing to the design, risk of bias, and small sample size.

**Conclusions:**

There is no definitive evidence about the relationship between Western dietary patterns and perinatal anxiety and depression. However, it found an inverse association among the healthy dietary pattern and PAAD. Future studies will be required to better evaluate associations between meal patterns and PAAD. Such studies may provide new insights and assist in the development of new prevention and treatment strategies.

## Background

The prenatal and postpartum periods represent highly vulnerable phases for the development of mental disorders owing to the significant physical, hormonal, and emotional changes that occur [1, 2]. Anxiety and depression are mental disorders highly prevalent in these periods and are associated with significant health consequences [2]. A recent systematic review study [3] reported that the prevalence of perinatal depression was 11.9%, whereas additional work showed the prevalence of prenatal and postpartum anxiety to be 15.2% [4] and 8.5% [5], respectively.

The specific factors contributing to the greater risk of PAAD include the absence of the partner or family support, a previous history of mental disorder or domestic violence, alcohol abuse, occurrence of pregnancy complications, changes estradiol and progesterone, and high intake of processed foods with excess sugar and fat content [1, 6–8]. One recent investigation showed that the perinatal consumption of a Western-type diet induced postpartum mood disorders, as well as depression-like phenotype and anxiety-like behavior in mice [9].

Several studies with pregnant women examined the association between nutritional factors and/or food with prenatal and postpartum anxiety and depression [10–12]. Data from these studies are important to the identification of specific nutritional factors associated with higher risk of or protection against PAAD. However, these investigations do not provide an adequate assessment of dietary exposure as the intake of individuals is complex and the bioavailability of various nutrients can be influenced by the presence or absence of many dietary factors [13, 14]. To address this disparity, epidemiological researchers have incorporated various statistical techniques to identify specific dietary patterns. This type of approach is essential to evaluate the association between diet (i.e., exposure) and disease (i.e., outcome) as it can address the variety, number, and combinations of foods and beverages consumed by individuals [13, 15, 16].

Assessment of dietary patterns and PAAD is particularly important as changes in eating habits often occur before, during and after pregnancy. During pregnancy, women adopt healthy eating habits to promote fetal development, as well as a better prognosis for childbirth and postpartum outcomes. However, some pregnant women exhibit unhealthy eating behaviors characterized by greater consumption of fast food*,* soft drinks, and other foods high in sugar and fat [17–19].

To date only one systematic review has been conducted to evaluate the association between diet/nutritional supplementation and perinatal depression. This review only included three studies that assessed dietary patterns and pregnancy [20]. There was also no discussion regarding the association between dietary patterns and perinatal anxiety.

The health consequences of PAAD include lower adherence to prenatal care, hypertension [21], increased health risk behaviors (e.g., alcohol and tobacco abuse) [22], and adverse neonatal effects (i.e., premature birth and low birth weight) [23, 24]. Thus, the ability to effectively assess of the relationship between dietary patterns and PAAD can represent a significant public health tool. As such, the objective of this systematic review is to evaluate the specific association between eating patterns and PAAD.

## Methods

This study adhered to PRISMA (Preferred Reporting Items for Systematic Reviews and Meta-Analyzes) [25]. The protocol of the systematic review was recorded in PROSPERO (International Register of Ongoing Prospective Systematic Reviews) (<http://www.crd.york.ac.uk/PROSPERO/>) with CRD 42017069097.

### Inclusion criteria

The inclusion criteria used were as follows: 1. cross-sectional and cohort studies without language restriction that included pregnant women, women in the 6 months before pregnancy or 6 months after delivery of any age group; 2. studies that utilized factor analysis, principal component analysis, cluster analysis, reduced rank regression, or treelet transform for the derivation of dietary patterns assessed 6 months before pregnancy and up to 6 months postpartum; and 3. studies that included an assessment of depression and/or anxiety during pregnancy or after delivery. The exclusion criteria used were review articles and studies using dietary indices or scores for the derivation of the dietary patterns.

### Search strategy

The virtual search was performed using PubMed, Scopus and LILACS databases. In PubMed and LILACS, the search strategy was: (diet OR diet pattern OR diet patterns OR dietary pattern OR dietary patterns OR eating pattern OR eating patterns OR food pattern OR food patterns) AND (posteriori OR factor analysis OR principal component factor analysis OR principal component analysis OR cluster analysis OR reduced rank regression OR treelet transform) AND (mental health OR mental problems OR mental disorder OR depress* OR anxiety) AND (pregnancy OR gestation OR pregnant OR postnatal OR postpartum OR perinatal OR ante-natal OR post-natal OR post-partum OR peri-natal). The search strategy used in the Scopus database was: TITLE-ABS-KEY (diet OR “diet pattern” OR “diet patterns” OR “dietary pattern” OR “dietary patterns” OR “eating pattern” OR “eating patterns” OR “food pattern” OR “food patterns”) AND TITLE-ABS-KEY (posteriori OR “factor analysis” OR “principal component factor analysis” OR “principal component analysis” OR “cluster analysis” OR “reduced rank regression” OR “treelet transform”) AND TITLE-ABS-KEY (“mental health” OR “mental problems” OR “mental disorder” OR depress* OR anxiety) AND TITLE-ABS-KEY (pregnancy OR gestation OR pregnant OR postnatal OR postpartum OR perinatal OR ante-natal OR post-natal OR post-partum OR peri-natal).

Two review authors (DS, SL) independently screened titles and abstracts from the articles identified through the database searches and subsequently assessed full texts articles to see if they met the eligibility criteria. Disagreements were resolved via consensus.

### Data extraction

The data extracted from the studies were entered in Table 1. Data included: name of the authors, year and language of publication, country where the study was conducted, study design, publication period of the article, number and age of participants, food surveys used to evaluate habitual consumption and corresponding statistical technique for the derivation of dietary patterns, identified dietary patterns, and association between dietary patterns and depression and anxiety.

**Table 1:** Characteristics of the studies included in the systematic review (*n* = 10)

Study characteristics		Participant characteristics		Exposure	Outcomes	Main results	Quality	
Study ID	Study design and Country	N / age	Gestational age during the selection period	Time assessed/ Dietary survey/ Methods of identification of dietary patterns/ identified dietary patterns	Time assessed/ Outcome/ Tool (Prevalence)	Associations between dietary patterns and depression and anxiety	NOS score	Global rating
Paskulin et al. (2017) [26]	Cross-sectional/ Brazil	712 / 24.6 ± 3.4 years	16 to 36 weeks	Pregnancy/ FFQ/ Cluster analysis/ Restricted, varied, and common-Brazilian pattern	Pregnancy/ Depression – PRIME-MD (21.6%) Anxiety – PRIME-MD (19.8%)	Common-Brazilian pattern (+) depression (RP = 1.62; 95% IC = 1.15, 2.30, *p* < 0.01). There was no statistically significant association between the restricted (western) and varied and anxiety during pregnancy.	7	Good
Baskin et al. (2017) [27]	Cohort / Australia	167 / 30.6 ± 4.3 years	10 to 16 weeks	Pregnancy/ FFQ/ Factor analysis/ Healthy, and unhealthy patterns	Pregnancy and post-partum/ Depressive symptoms–EPDS (28%)	Unhealthy pattern identified in 32.89 (SD = 0.89) week of gestation: (+) Depressive symptoms in 16.70 (SD = 0.91) week of gestation = (β = 0.17, 95% CI = 0.32, 0.02, *p* < 0.05) and in 32.89 (SD = 0.89)) week of gestation = (β = 0.19, 95% CI = 0.04, 0.34, *p* < 0.05). There was no statistically significant association between healthy pattern with pregnancy and postpartum depressive symptoms, and between unhealthy pattern and postpartum depressive symptoms.	8	Good
Vilela et al. (2015) [28]	Cohort / Brazil	207 / 20 to 40 years	5 to 13 weeks	6 months prior to pregnancy/ FFQ/ Factor analysis/ common-Brazilian, healthy, and processed (western) patterns	Second and third pregnancy trimesters and at postpartum/ Anxiety/ STAI (second trimester: 40.4%, third trimester: 40.5% and postpartum: 37.2%.	Common-Brazilian pattern: (−) postpartum anxiety (β = − 1200, 95% CI = − 2.220, − 0.181, *P* < 0.02). Healthy pattern: (−) postpartum anxiety (β = − 1290, 95% CI = − 2438, − 0,134, *P* < 0.03). There was no statistically significant association between common-Brazilian and healthy patterns and anxiety in pregnancy, and the processed (western) pattern and pregnancy and postpartum anxiety.	8	Good
Vilela et al. (2014) [29]	Cohort / Brazil	248 / 26.7 years	5 to 13 weeks	6 months prior to pregnancy/ FFQ/ Factor analysis/ common-Brazilian, healthy, and processed (western) patterns.	Pregnancy/ Depressive symptoms –EPDS (not reported)	Healthy pattern: (−) Depressive symptoms (β = − 0.723, 95% CI: − 1.277, − 0.169, *P* = 0.011). There was no statistically significant association between common-Brazilian and processed (western) patterns and depressive symptoms in pregnancy.	8	Good
Vaz et al. (2013) [30]	Cross-sectional/ United Kingdom	9.530 / < 25 years: 2.078 ≥25 years: 7.452	32nd week of gestation	Pregnancy/ FFQ/ Factor analysis/ Health-conscious, traditional, processed, confectionery, and vegetarian patterns.	Pregnancy/ Anxiety/ CCEI/ < 25 years: 21.9%; ≥25 years: 14.6%.	Health conscious pattern: (−) anxiety (OR = 0.77; 95% CI = 0.65, 0.93, *P* < 0.01). Traditional pattern: (−) anxiety (OR = 0.84; 95% IC = 0.73, 0.97, *P* < 0,01). Confectionery pattern: (+) anxiety (OR = 1.24; 95% CI = 1:06, 1:45, *P* < 0.01), not in the model adjusted for maternal intake of n-3 PUFA fatty acids. Vegetarian Pattern: (+) anxiety (OR = 1.25; 95% CI: 1.08, 1.44, *P* < 0.01). There was no statistically significant association between processed pattern and anxiety in pregnancy.	7	Good
Okubo et al. (2011) [31]	Cohort / Japan	865 / 29.9 ± 4.0 years	20th week of gestation	Pregnancy/ Food history/ Factor analysis/ Healthy, western, and Japanese patterns.	Postpartum/ Depressive symptoms –EPDS (14%)	There was no statistically significant association between healthy, western and Japanese patterns and postpartum depressive symptoms.	7	Good
Chatzil et al. (2011) [32]	Cohort / Greece	529 / no presented	6th month	Pregnancy/ FFQ/ Factor analysis/ Western, and health conscious.	Postpartum/ depressive symptoms –EPDS (14%)	Health conscious pattern: (−) depressive symptoms –third tertile versus lowest tertile of the ‘health conscious’ dietary pattern (RR = 0.51, 95% CI 0.25, 1.05). There was no statistically significant association between western pattern and postpartum depressive symptoms.	7	Good
Maracy et al. (2014) [33]	Cross-sectional/ Iran	770	10 days to 3 months postpartum	Evaluated in postpartum but referring to gestation / FFQ/ Factor analysis/ Mixed, semi-healthy and fruits, and vegetables patterns.	Postpartum/ Depressive symptoms– EPDS/ 34.6%	Semi-healthy dietary pattern: (−) Depressive symptoms (OR = 0.60, 95%CI: 0.38, 0.94; *P* = 0.05). Fruit and vegetable pattern: (−) Depressive symptoms (OR = 0.52, 95%CI: 0.32, 0.84; *P* = 0.004). There was no statistically significant association between mixed pattern with postpartum depressive symptoms.	7	Good
Teo et al. (2018) [34]	Cohort / Singapure	490 / 31.4 ± 4.8	26 to 28 weeks	Evaluated in postpartum (3 weeks post-delivery)/ 3-day food diaries/ Factor analisys/ Traditional-Chinese-Confinement, Traditional-Indian-Confinement, The Eat-Out diet, and Soup-Vegetables-Fruits.	Three months postpartum/ Depression and anxiety/ EPDS and STAI	Traditional-Indian-Confinement diet (depressive symptoms) = − 0.62 EPDS scores per SD increase in TIC score; 95% CI = − 1.16, − 0.09), *p* = 0.02. There was no statistically significant association between Traditional-Chinese-Confinement, The Eat-Out diet, and Soup-Vegetables-Fruits with depression. Soup-Vegetables-Fruits (−) anxiety (− 1.49 STAI-state subscale scores per SD increase in SVF score; 95% CI = − 2.56, − 0.42), *p* = 0.006). There was no statistically significant association between Traditional-Chinese-Confinement, Traditional-Indian-Confinement, The Eat-Out diet with anxiety.	8	Good
Miyakea et al. (2018) [35]	Cross-sectional/Japan	1744/ 31.2 ± 4.3	5 to 39 weeks	Pregnancy/ DHQ/ Factor analysis/ Healthy, Japanese, and Westernpattern	Pregnancy/ Depressive symptoms/CES-D scale/ 19.2%	Japanese pattern (−) Depressive symptoms (third vs first quartile – PRs = 0.76; 95% CIs = 0.58–0.998, P for trend = 0.008 and fourth vs first quartile – PRs = 0.72; 95% CIs = 0.55–0.94, P for trend = 0.008). Healthy pattern (−) Depressive symptoms (second vs first quartile – PRs = 0.70; 95% CIs = 0.55–0.89; *P* < 0.0001; third vs first quartile – PRs = 0.48; 95% CIs = 0.36–0.64; *P* < 0.0001, and fourth vs first quartile – PRs = 0.56; 95% CIs = 0.43–0.73; *P* < 0.0001). There was no statistically significant association between the western patternand depressive symptomsduring pregnancy.	5	Good

*Abbreviations*: *95%CI*, 95% confidence interval, *CCEI* Crown-Crisp Experiential Index, *CES-D* Center for Epidemiologic Studies Depression Scale, *DHQ* Diet History Questionnaire, *EPDS* Edinburgh Postnatal Depression Scale, *FFQ* Food frequency questionnaire, *NOS* Newcastle-Ottawa Scale, *OR* Odds ratio, *PR* Prevalence ratio, *PRIME-MD* Primary Care Evaluation of Mental Disorders, *STAI* State-Trait Anxiety Inventory

### Methodological quality

Assessment of methodological quality and risk of bias in the primary study with cohort design was performed using NOS [36], which included eight items related to selection, comparison, and outcome. For each item a star is awarded (except for comparison) that can receive up to two stars. The studies with six stars (maximum of nine) were classified as good quality. For cross-sectional studies, we used a modified version of NOS [37] in which studies that received at least five stars (maximum of eight) were classified as good quality.

### Best-evidence synthesis

Summary of evidence was provided for associations between western, healthy and traditional dietary patterns with PAAD. Considering that studies often present different definitions for dietary patterns with a similar food composition, we defined the composition of the Western dietary pattern (independent of the denomination given in the original study) as a diet involving the high intake of meats, processed cereals, high fat foods, and products with high sugar content. In contrast, a healthy dietary pattern consists predominantly of whole foods, fruits, vegetables, fish and seafood, and skimmed milk products. Finally, a traditional dietary patterns was defined as those described by the typical food consumption patterns of the studied regions.

The GRADE framework was used to assess the quality of evidence in regard to the association between dietary patterns with PAAD [38, 39]. GRADE ranks the evidence as: 1. high (there is strong certainty that the association is close to the estimated); 2. moderate (there is moderate certainty in the estimated association); 3. low (certainty in association is limited); and 4. very low (certainty in the association estimate is very limited owing to a significant degree of uncertainty in the findings). The evaluation criteria used for outcomes included the design in which clinical trials were given an A and observational studies a C. The high estimate of the association and overall consistency of the findings also affected the grade. Additionally, the presence of relevant methodological limitations, indirect evidence, and publication bias were all contributing factors that affected the score [38, 39].

## Results

### Study characteristics

**Fig. 1:**
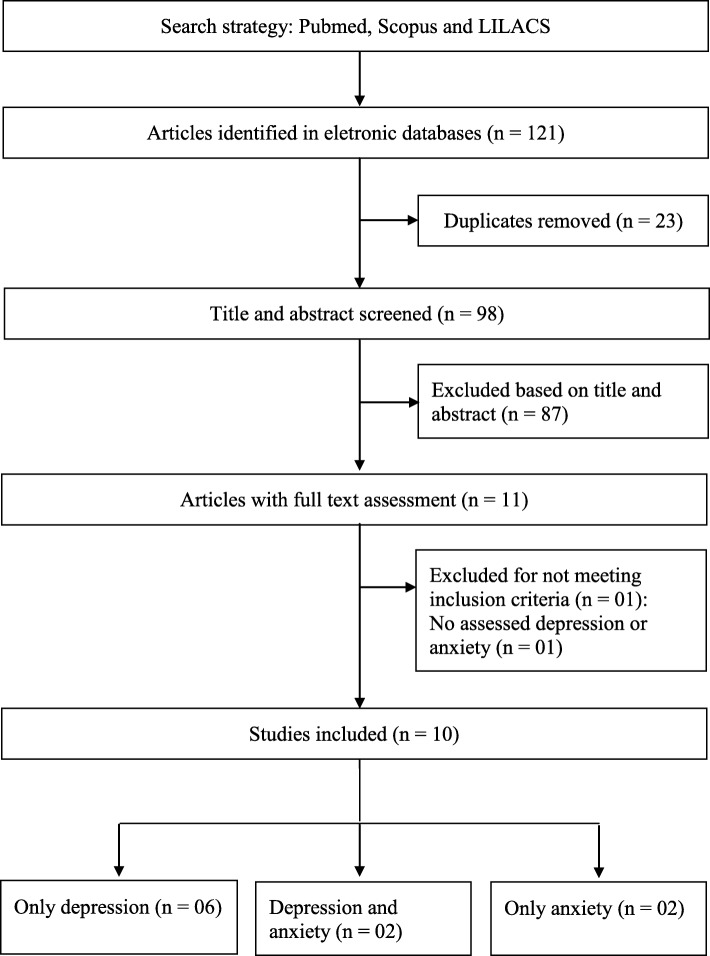
Process used in the selection of articles

The virtual searches recovered a total of 121 studies (90 from PubMed, 31 from Scopus, and 0 from LILACS*.* There were 23 duplicate records that were excluded, which resulted in 98 abstracts. Following evaluation of the title and abstract, an additional 87 articles were excluded. For the 11 studies that had full-text analysis, 10 met this study’s eligibility criteria and were subsequently included in the review [26–35]. The flowchart of the selection process is presented in Fig. 1.

The characteristics of included studies are presented in Table 1. The number of participants in each study ranged from 167 [27] to 9530 [30]. The selection period for included studies ranged from 5 weeks of gestation [28, 29, 35] to 3 months postpartum [33].

### Assessment of dietary pattern characteristics

Six studies examined usual dietary intake during pregnancy [26, 27, 30–32, 35]. Two studies [28, 29] evaluated 6 months prior to pregnancy, whereas two other studies evaluated in the postpartum period [33, 34]. The survey methods used included a food frequency questionnaire (*n* = 7) [26–30, 32, 33], food history questionnaire (*n* = 2) [31, 35], and 3-day food record (*n* = 1) [34]. For statistical analysis, nine studies used factor analysis [27–35] and one used cluster analysis for evaluation of dietary pattern [26].

The 10 studies included in this review identified food consumption patterns characterized by a Western pattern [26–35], which was referenced as processed (*n* = 3) [28–30], Western (*n* = 3), [31, 32, 35]^,^ restricted (*n* = 1) [26], unhealthy (*n* = 1) [27], confectionery (*n* = 1) [30], mixed (*n* = 1) [33], or Eat-Out diet (*n* = 1) [34]. Nine studies identified dietary patterns classified as healthy patterns [27–35], whereas traditional patterns were identified in seven studies [26, 28–31, 34, 35] conducted in Brazil (*n* = 3) [26, 28, 29], the United Kingdom (*n* = 1) [30], Japan (*n* = 2) [31, 35], and Singapore (*n* = 1) [34]. The food composition for western, healthy, and traditional dietary patterns is presented in Table 2.

**Table 2:** Characteristics of the dietary patterns

Study ID	Dietary patterns (% adesion)
Paskulin et al. (2017) [26]	Restricted dietary pattern (205 women [28.8%]):Cookies, snacks, French fries, soft drinks, whole milk, natural juice, yogurt, cocoa powder, and ice cream. Varied pattern (244 women [34.3%]):wide variety of foods from the following groups: cereals, grains, and tubers; cakes, breads, and cookies; fruits and vegetables. Common-Brazilian pattern (263 women [36.9%]): beans, rice or noodles, boneless beef/chicken or eggs, French rolls, margarine, coffee with sugar, and artificial juices.
Baskin et al. (2017) [27]	Healthy pattern: vegetables, fruit, nuts, fish and seafood, eggs, whole grainswater, and tea. Unhealthy pattern: condiments, refined grains, sweets and desserts, fast foods, highenergy drinks, high-fat dairy, hot chips, fruit juice and red meats and high negative loadings on oil/vinegar-based and nuts-based dressing.
Vilela et al. (2015) [28]	Common-Brazilian pattern: beans, rice, meats and eggs, and vegetable spices. Healthy pattern: roots and tubers, legumes and green vegetables, fruits and fruit juice, fish, dairy products, pasta, tea, cakes, cookies-crackers, and candies, and was inversely related with coffee intake. Processed pattern:bread, fat, sugar, snacks and fast food, soft drinks, and deli meats and sausages.
Vilela et al. (2014) [29]	Common-Brazilian pattern: Rice, beans, meats, eggs, vegetable spices. Healthy pattern: Dairy products, green vegetables and legumes, fruits and fruit juices, fish, candies, noodles, cakes and cookies/crackers, pasta, roots, tubers, tea. Processed pattern: Bread, fast food and snacks, fat, sausages and deli meats, sugar, soft drinks, coffee.
Santos Vaz et al. (2013) [30]	Healthconscious pattern’: Salad, fruit, fruit juice, fish, pasta, rice, oat/bran based breakfast cereal, cheese, pulses, non-white bread. Traditional pattern: Vegetables, red meat, poultry. Processed pattern: Meat pies, burgers, sausages, fried foods, chips, pizza, eggs, white bread, baked beans. Confectionery pattern: Chocolate, biscuits, sweets, puddings, cakes. Vegetarian pattern: Meat substitutes, pulses, herbal, nuts, tea and high negative loadings for red meat and poultry.
Okubo et al. (2011) [31]	Healthy pattern: Green and yellow vegetables, white vegetables, potatoes, seaweeds, fruits, fish, shellfish and sea products. Western pattern: Vegetable oil, beef and pork, processed meat, chicken and eggs, salt-containing seasonings. Japanese pattern: Rice, sea products, miso soup, fish and pickled vegetables.
Chatzil et al. (2011) [32]	Western pattern: Meat and meat products, sugar and sweets, potatoes, fats except olive oil, cereals, eggs, salty snacks, beverages and sauces. Health conscious pattern: Vegetables, fruit, pulses, nuts, olive oil, fish and seafood, and dairy products.
Maracy et al. (2014) [33]	Mixed pattern: Legumes, red meat, salt, oil, processed meat, offal, chicken, fish, potato, eggs, snacks, sugar, nuts and refined grains. Semi-healthy pattern: Fruits, juices and sweets desserts, butter, nuts, processed cereals, pickles, low-fat dairy products, fruit jelly, pasta and dairy products. Fruits and vegetables pattern: Cabbage, green vegetables, green leaves, other vegetables, fruits, tomatoes. Variance explained: 41%.
Teo et al. (2018) [34]	The Traditional-Chinese-Confinement (TCC) diet: Characterized by high consumption of traditional dried fruits, Chinese herbs, rhizomes, herbal tea, and foods cooked with alcohol, wine, or vinegar. The Traditional-Indian-Confinement (TIC) diet: Compost by ethnic bread, whole milk, Indian herbs, seed herbs, and butter/ghee. The Eat-Out diet: Characterized by high consumption of deep-fried/mashed potato, ice-cream, sweetened and cordial drinks, deep-fried dimsum, chips/crisps, and local savoury snacks. Soup-Vegetables-Fruits (SVF) diet: Higher intakes of assorted soup (vegetables, seafood, fish, meat, and noodles), fish (non-fried), vegetables, and fresh fruits, and lower intakes of milk-based drinks and sweet spreads.
Miyakea et al. (2018) [35]	Healthy pattern (10.1%): Green and yellow vegetables, other vegetables, pulses, miso soup, mushrooms, seaweed, fish, potatoes, sea products, sugar, and shellfish and a low intake of bread andconfectioneries. Japanese pattern (6.2%): High intake of rice and misosoup and low intake of coffee and cocoa, dairy products, confectioneries, sugar, and bread. Western pattern (5.8%):High intake of processed meat, beef and pork, chicken, vegetable oil, shellfish, eggs, and salt-containing seasonings and a low intake of bread.

### Assessment of anxiety and depression characteristics

Assessment of prenatal (*n* = 2) [26, 30], postpartum (*n* = 1) [34], and perinatal (during pregnancy and postpartum – included 30 to 45 days after delivery; *n* = 1) [28] anxiety was carried out by four studies. The criteria used for evaluation of anxiety included the State-Trait Anxiety Inventory [28, 34], Primary Care Evaluation of Mental Disorders (PRIME-MD) [26], and Crown-Crisp Experiential Index [30]. Assessment of prenatal (*n* = 3) [26, 29, 35], postpartum (8 weeks to 9 months; *n* = 4) [31–34], and perinatal (*n* = 1) [27] depression/depressive symptoms was conducted in eight studies [27]. The criteria used for the evaluation of depression included the PRIME-MD (*n* = 1) [26], and Center for Epidemiologic Studies Depression Scale (*n* = 1) [35]. The Edinburgh Postnatal Depression Scale (*n* = 6) [27, 29, 31–34], was used for evaluation of depressive symptoms.

### Quality assessment

All cohort studies included in the review showed higher scores than six stars on the NOS scale, which is classified as having good methodological quality [27–29, 31, 32, 34]. The cross-sectional studies also exhibited good quality as observed scores were greater than or equal to five stars [26, 30, 33, 35].

### Summary of evidence

The summary of the associations between dietary patterns and PAAD is presented in Table 3.

**Table 3:** Summary of evidence of the associations between dietary patterns identified in the studies with perinatal anxiety and depression

Exposure	Outcomes	Participants (studies)	Quality of evidence (GRADE)	Evidence summary
Western pattern	Prenatal depression	2.871 (4)	⊕●●● Very low due to number of studies, design and bias.	No association
Healthy pattern	Prenatal depression	2.159 (3)	⊕●●● Very low due to number of studies, design and bias.	Negative association
Traditional Brazilian pattern	Prenatal depression	960 (2)	–	Inconclusive result
Traditional Japanese pattern	Prenatal depression	1.744 (1)	⊕●●● Very low due to number of studies, design and bias.	Negative association
Western pattern	Postpartum depression	2.821 (5)	⊕⊕●● Low because to heterogeneity	No association
Healthy pattern	Postpartum depression	2.821 (5)	–	Inconclusive result
Japanese traditional pattern	Postpartum depression	865 (1)	⊕●●● Very low due to number of studies, design and bias.	No association
Traditional-Chinese-Confinement (TCC) pattern	Postpartum depression	490 (1)	⊕●●● Very low due to number of studies, design and bias.	No association
Traditional-Indian-Confinement pattern	Postpartum depression	490 (1)	⊕●●● Very low due to number of studies, design and bias.	Negative association
Western pattern	Prenatal anxiety	10.242 (2)	–	Inconclusive result
Healthy pattern	Prenatal anxiety	9.530 (1)	⊕●●● Very low due to number of studies, design and bias.	Negative association
Traditional Brazilian pattern	Prenatal anxiety	712 (1)	⊕●●● Very low due to number of studies, design and bias.	No association
United Kingdom’ Traditional pattern	Prenatal anxiety	9.530 (1)	⊕●●● Very low due to number of studies, design and bias.	Negative association
Western pattern	Postpartum anxiety	697 (2)	⊕●●● Very low due to number of studies, design and bias.	No association
Healthy pattern	Postpartum anxiety	697 (2)	⊕●●● Very low due to number of studies, design and bias.	Negative association
Traditional Brazilian pattern	Postpartum anxiety	207 (1)	⊕●●● Very low due to number of studies, design and bias.	Negative association
Traditional-Chinese-Confinement (TCC) pattern	Postpartum anxiety	490 (1)	⊕●●● Very low due to number of studies, design and bias.	No association
Traditional-Indian-Confinement pattern	Postpartum anxiety	490 (1)	⊕●●● Very low due to number of studies, design and bias.	No association

### Association between dietary patterns and prenatal depressive symptoms / depression

A Western dietary pattern was positively associated with prenatal depressive symptoms in one study [27], whereas three other studies [26, 29, 35] reported no statistically significant association. The summary of evidence demonstrated very low quality based on the GRADE framework. In contrast, a healthy dietary pattern was negatively associated with prenatal depressive symptoms/depression in two studies [29, 35]. No other study reported a statistically significant association with a healthy dietary pattern and prenatal depressive symptoms [27]. The summary of evidence also demonstrated very low quality based on the GRADE framework.

There was no significant association between traditional Brazilian dietary pattern and prenatal depressive symptoms/depression in two studies [26, 29]. Another study reported a negative association between traditional Japanese dietary pattern and prenatal depression [35],. with very low quality of evidence based on the GRADE framework.

### Association between dietary patterns and postpartum depression

The five studies that evaluated the association between a Western dietary pattern and postpartum depression reported no statistically significant association [27, 31–34]. The summary of evidence demonstrated very low quality based on the GRADE framework. The summary of evidence for the association between a healthy dietary pattern and postpartum depression was evaluated in five studies, but was reported to be inconclusive owing to divergences in the findings [27, 31–34].

A study by Okubo et al. evaluated the association between a traditional Japanese dietary pattern and postpartum depression. No statistically significant association was observed and the summary of evidence showing very low quality based on the GRADE framework. A traditional Indian-confinement dietary pattern was negatively associated with postpartum depression, whereas a traditional Chinese-confinement dietary pattern reported no statistically significant association with the associations from each traditional dietary pattern exhibiting very low evidence quality, based on the GRADE framework [34].

### Association between dietary patterns and prenatal anxiety

The summary of evidence for the association between a Western dietary pattern and prenatal anxiety was inconclusive. However, a positive association was found in one study [30], whereas another study found no statistically significant association [26]. A healthy dietary pattern was negatively associated with prenatal anxiety in one study, although the summary of evidence demonstrated very low quality based on the GRADE framework [30]. The relationship between anxiety and a traditional Brazilian dietary pattern showed no significant association [26]. A study that examined the association with a United Kingdom traditional dietary pattern reported a negative association, although the summary of evidence demonstrated very low quality based on the GRADE framework [30].

### Association between dietary patterns and postpartum anxiety

Among the included studies, two evaluated the association between dietary patterns and postpartum anxiety [28, 34]. A healthy dietary pattern [28, 34] and a traditional Brazilian dietary pattern [28] were both reported to be negatively associated with postpartum anxiety. The summary of evidence for both associations was very low-quality based on the GRADE framework. In contrast, there was no significant association between a Western dietary pattern, a traditional Indian confinement dietary pattern, or traditional-Chinese confinement dietary pattern and postpartum anxiety. The summary of evidence for all three associations was reported as very low quality [34].

## Discussion

All studies included in this review identified the unhealthy/Western style dietary pattern, characterized by the inclusion of processed meat, refined grains, high-fat foods, and products with high sugar content [26–35]. This food composition of the Western dietary pattern is quite different from the recommendations for prenatal and postpartum nutrition [40]. This is of concern as perinatal periods have greater nutritional requirements to protect against adverse events for women and the fetuses/neonates. In particular, gestational diabetes, preeclampsia, reduced fetal growth, and low birth weight are of major concern. Several studies reported a positive association between a western dietary pattern and these adverse events [41–43].

The summary of evidence of the association between a Western dietary pattern and perinatal anxiety and depressive symptoms/depression showed inconclusive findings or no significant association. These results differ from previously systematic reviews that reported that a western dietary pattern was associated with greater risks of developing depression-related symptoms [44, 45].

A recent meta-analysis by Li et al. was based on 21 studies and revealed a positive association between the Western dietary pattern and depression (i.e., higher risk for depression). However, this analysis [45] only included one study focused on pregnant women [31].

Dietary patterns that constituted of whole foods, fruits, vegetables, fish and seafood were characterized as a healthy dietary pattern. Unfortunately, these dietary patterns typically have lower adherence compared to a Western dietary pattern [46]. A healthy diet pattern is associated with positive outcomes for pregnant women, as well as fetuses and neonates. This is evident from a lower risk of gestational diabetes and premature labor [47, 48].

A healthy dietary pattern was negatively associated with prenatal and postpartum anxiety and depression [27–30, 34, 35]. This was verified by additional systematic reviews that included various age groups that showed health dietary pattern is associated with reduced risk of depression [44, 45].

The dietary patterns that represent the normal food from each country were identified as a traditional dietary pattern. Five traditional patterns were identified: 1. Brazilian (composed in general of beans, rice, beef and eggs) [26, 28, 29]; 2. United Kingdom (included vegetables, beef and chicken) [30]; 3. Japanese (consisting of rice, miso soup, fish and seafood and pickled vegetables) [31, 35]; 4. Traditional-Chinese-Confinement (characterized by consumption of traditional dried fruits, Chinese herbs, rhizomes, herbal tea, and foods cooked with alcohol, wine, or vinegar) [34]; and 5. Traditional-Indian-Confinement (composed of ethnic bread, whole milk, Indian herbs, seed herbs, and butter/ghee). These last two dietary patterns are commonly observed in Singapore [34].

We emphasize the adherence to traditional dietary patterns for each country rather than an unhealthy or western dietary pattern, which is the result of a global food transition that has recently become more apparent [49]. An increased concern regarding this global food transition is justified as the changes typically result in the inclusion and/or greater consumption of foods with high sugar, fat and sodium [49].

The traditional Japanese dietary pattern and a traditional dietary pattern in Singapore were negatively associated with prenatal and postpartum depression, respectively [34, 35], whereas prenatal and postpartum anxiety were negatively associated with a United Kingdom traditional dietary pattern and traditional Brazilian dietary pattern, respectively [28, 30].

There are multiple factors influencing the development of anxiety and depressive symptoms, which may explain the inconclusive and non-significant associations.

The limitations of this systematic review include 1. heterogeneity among the included studies; 2. different instruments used for the diagnosis of depression / assessment of depressive symptoms and anxiety; 3. variation in the participants and follow-up time for pregnant women; 4. lack of standardization for the definition of dietary patterns. Because of these limitations a meta-analysis could not be used on these data.

Despite these limitations, the evaluation of the methodological quality using specific criteria for cross-sectional and cohort studies, as well as the evaluation of the quality of evidence by the GRADE framework are beneficial outcomes from this study. To our knowledge, this is the first systematic review to exclusively evaluate the association between PAAD and dietary patterns of pregnant women defined using factor analysis or cluster analysis.

## Conclusion

There is no definitive evidence about the relationship between Western dietary patterns and perinatal anxiety and depression. However, it found an inverse association among the healthy dietary pattern and PAAD. The traditional Japanese dietary pattern and the traditional-Indian-Confinement dietary pattern were negatively associated with postpartum depression. The United Kingdom traditional dietary pattern was negatively associated with prenatal anxiety, whereas the traditional Brazilian dietary pattern was negatively associated with postpartum anxiety. More high quality studies will be required fully elucidate this relationship, which has significant implications for maternal and fetal health and well-being.

## Data Availability

All data generated or analyzed in this study are included in the article.

## Abbreviations

GRADE: Grading of Recommendations Assessment, Development and Evaluation
LILACS: Latin American & Caribbean Health Sciences Literature
NOS: Newcastle-Ottawa scale
PAAD: Perinatal anxiety and depression
PRISMA: Preferred Reporting Items for Systematic Reviews and Meta-Analyzes

## References

[1] Biaggi A, Conroy S, Pawlby S, Pariante CM (2016). Identifying the women at risk of antenatal anxiety and depression: a systematic review. J Affect Disord.

[2] Fisher J, Mello MCD, Patel V (2012). Prevalence and determinants of common perinatal mental disorders in women in low-and lower-middle-income countries: a systematic review. Bull World Health Org.

[3] Woody CA, Ferrari AJ, Siskind DJ (2017). A systematic review and meta-regression of the prevalence and incidence of perinatal depression. J Affect Disord.

[4] Dennis CL, Falah-Hassani K, Shiri R (2017). Prevalence of antenatal and postnatal anxiety: systematic review and meta-analysis. Br J Psychiatry.

[5] Goodman JH, Watson GR, Stubbs B (2016). Anxiety disorders in postpartum women: a systematic review and meta-analysis. J Affect Disord.

[6] Schiller CE, Meltzer-Brody S, Rubinow DR (2015). The role of reproductive hormones in postpartum depression. CNS Spectr.

[7] Pina-Camacho L, Jensen SK, Gaysina D (2015). Maternal depression symptoms, unhealthy diet and child emotional–behavioural dysregulation. Psychol Med.

[8] Baskin R, Hill B, Jacka FN (2015). The association between diet quality and mental health during the perinatal period. A systematic review. Appetite..

[9] Bolton JL, Wiley MG, Ryan B (2017). Perinatal western-type diet and associated gestational weight gain alter postpartum maternal mood. Brain Behav.

[10] Chong MF, Wong JX, Colega M (2014). Relationships of maternal folate and vitamin B12 status during pregnancy with perinatal depression: the GUSTO study. J Psychiatr Res.

[11] Rocha CM, Kac G (2012). High dietary ratio of omega-6 to omega-3 polyunsaturated acids during pregnancy and prevalence of post-partum depression. Matern Child Nutr..

[12] Lewis SJ, Araya R, Leary S (2012). Folic acid supplementation during pregnancy may protect against depression 21 months after pregnancy, an effect modified by MTHFR C677T genotype. Eur J Clin Nutr.

[13] Barkoukis H (2007). Importance of understanding food consumption patterns. J Acad Nutr Diet.

[14] Wirfält E, Drake I, Wallström P (2013). What do review papers conclude about food and dietary patterns?. Food Nutr Res.

[15] Jacques PF, Tucker KL (2001). Are dietary patterns useful for understanding the role of diet in chronic disease?. Am J Clin Nutr.

[16] Slattery ML (2010). Analysis of dietary patterns in epidemiological research. Appl Physiol Nutr Metab.

[17] Gomes CB (2015). Práticas alimentares de gestantes e mulheres não grávidas: há diferenças?. Rev Bras Ginecol Obstet.

[18] Soares RM, Nunes MA, Schmidt MI (2009). Inappropriate eating behaviors during pregnancy: prevalence and associated factors among pregnant women attending primary care in southern Brazil. Int J Eat Disord.

[19] Czech-Szczapa B, Szczapa T, Merritt TA (2015). Disordered eating attitudes during pregnancy in mothers of newborns requiring neonatal intensive care unit admission: a case control study. J Matern Fetal Neonatal Med.

[20] Sparling TM, Henschke N, Nesbitt RC (2017). The role of diet and nutritional supplementation in perinatal depression: a systematic review. Matern Child Nutr.

[21] Thombre MK, Talge NM, Holzman C (2015). Association between pre-pregnancy depression/anxiety symptoms and hypertensive disorders of pregnancy. J Women's Health.

[22] Chan J, Natekar A, Einarson A (2014). Risks of untreated depression in pregnancy. Can Fam Physician.

[23] Szegda K, Markenson G (2014). Bertone-Johnson, et al. depression during pregnancy: a risk factor for adverse neonatal outcomes? A critical review of the literature. J Matern Fetal Neonatal Med.

[24] Grigoriadis S, VonderPorten EH, Mamisashvili L (2013). The impact of maternal depression during pregnancy on perinatal outcomes: a systematic review and meta-analysis. J Clin Psychiatry.

[25] Moher D, Liberati A, Tetzlaff J (2009). Preferred reporting items for systematic reviews and meta-analyses: the PRISMA statement. PLoS Med.

[26] Paskulin JT, Drehmer M, Olinto MT (2017). Association between dietary patterns and mental disorders in pregnant women in southern Brazil. Rev Bras Psiquiatr.

[27] Baskin R, Hill B, Jacka FN (2017). Antenatal dietary patterns and depressive symptoms during pregnancy and early post-partum. Matern Child Nutr..

[28] Vilela AAF, Pinto JPT, Rebelo F (2015). Association of prepregnancy dietary patterns and anxiety symptoms from midpregnancy to early postpartum in a prospective cohort of Brazilian women. J Acad Nutr Diet.

[29] Vilela AAF, Farias DR, Eshriqui I (2014). Prepregnancy healthy dietary pattern is inversely associated with depressive symptoms among pregnant Brazilian women. J Nutr.

[30] Santos Vaz J, Kac G, Emmett P (2013). Dietary patterns, n-3 fatty acids intake from seafood and high levels of anxiety symptoms during pregnancy: findings from the Avon longitudinal study of parents and children. PLoS One.

[31] Okubo H, Miyake Y, Sasaki S (2011). Dietary patterns during pregnancy and the risk of postpartum depression in Japan: the Osaka maternal and child health study. Br J Nutr.

[32] Chatzi L, Melaki V, Sarri K (2011). Dietary patterns during pregnancy and the risk of postpartum depression: the mother–child ‘Rhea’cohort in Crete, Greece. Public Health Nutr.

[33] Maracy MR, Iranpour S, Esmaillzadeh A (2014). Dietary Patterns During Pregnancy and the Risk of Postpartum Depression. Iran J Epidemiol.

[34] Teo C, Chia AR, Colega M (2018). Prospective associations of maternal dietary patterns and postpartum mental health in a multi-ethnic asian cohort: the growing up in Singapore towards healthy outcomes (gusto) study. Nutrients..

[35] Miyake Y, Tanaka K, Okubo H (2018). Dietary patterns and depressive symptoms during pregnancy in Japan: baseline data from the Kyushu Okinawa maternal and child health study. J Affect Disord.

[36] Wells GA, Shea B, O’Connell D, et al. The Newcastle-Ottawa scale (NOS) for assessing the quality of nonrandomised studies in meta-analyses. http://www.ohri.ca/programs/clinical_epidemiology/oxford.asp. Accessed 20 Dec 2017.

[37] The modified Newcastle Ottawa scale for cross sectional studies. http://journals.plos.org/plosone/article/file?id=info%3Adoi/10.1371/journal.pone.0136065.s004&type=supplementary. Accessed 20 Dec 2017.

[38] Guyatt G, Vist G, Falck-Ytter Y (2006). An emerging consensus on grading recommendations?. Evid Based Med.

[39] Guyatt GH, Oxman AD, Vist GE (2008). GRADE: an emerging consensus on rating quality of evidence and strength of recommendations. BMJ..

[40] Procter SB, Campbell CG (2014). Position of the academy of nutrition and dietetics: nutrition and lifestyle for a healthy pregnancy outcome. J Acad Nutr Diet.

[41] Zhang C, Ning Y (2011). Effect of dietary and lifestyle factors on the risk of gestational diabetes: review of epidemiologic evidence. Am J Clin Nutr.

[42] Schoenaker DA, Soedamah-Muthu SS, Mishra GD (2014). The association between dietary factors and gestational hypertension and pre-eclampsia: a systematic review and meta-analysis of observational studies. BMC Med.

[43] Okubo H, Miyake Y, Sasaki S (2012). Maternal dietary patterns in pregnancy and fetal growth in Japan: the Osaka maternal and child health study. Br J Nutr.

[44] Rahe C, Unrath M, Berger K (2014). Dietary patterns and the risk of depression in adults: a systematic review of observational studies. Eur J Nutr.

[45] Li Y, Lv MR, Wei YJ (2017). Dietary patterns and depression risk: a meta-analysis. Psychiatry Res.

[46] Knudsen VK, Orozova-Bekkevold IM, Mikkelsen TB (2008). Major dietary patterns in pregnancy and fetal growth. Eur J Clin Nutr.

[47] Englund-Ögge L, Brantsæter AL, Sengpiel V (2014). Maternal dietary patterns and preterm delivery: results from large prospective cohort study. BMJ..

[48] Tobias DK, Zhang C, Chavarro J (2012). Prepregnancy adherence to dietary patterns and lower risk of gestational diabetes mellitus. Am J Clin Nutr.

[49] Popkin BM, Adair LS, Ng SW (2012). Global nutrition transition and the pandemic of obesity in developing countries. Nutr Rev.

